# Development of a Fish Cell Biosensor System for Genotoxicity Detection Based on DNA Damage-Induced Trans-Activation of *p21* Gene Expression

**DOI:** 10.3390/bios2030318

**Published:** 2012-09-10

**Authors:** Deyu Geng, Zhixia Zhang, Huarong Guo

**Affiliations:** Department of Marine Biology, Ocean University of China, Qingdao 266003, China; E-Mails: doarey@126.com (D.G.); zhangzhixia_0519@yahoo.com.cn (Z.Z.)

**Keywords:** genotoxicity, fish cells, *p21*, *p53*, trans-activation, luciferase

## Abstract

*p21*^CIP1/WAF1^ is a *p53*-target gene in response to cellular DNA damage. Here we report the development of a fish cell biosensor system for high throughput genotoxicity detection of new drugs, by stably integrating two reporter plasmids of pGL_3_-p21-luc (human *p21* promoter linked to firefly luciferase) and pRL-CMV-luc (CMV promoter linked to *Renilla* luciferase) into marine flatfish flounder gill (FG) cells, referred to as p21FGLuc. Initial validation of this genotoxicity biosensor system showed that p21FGLuc cells had a wild-type *p53* signaling pathway and responded positively to the challenge of both directly acting genotoxic agents (bleomycin and mitomycin C) and indirectly acting genotoxic agents (cyclophosphamide with metabolic activation), but negatively to cyclophosphamide without metabolic activation and the non-genotoxic agents ethanol and D-mannitol, thus confirming a high specificity and sensitivity, fast and stable response to genotoxic agents for this easily maintained fish cell biosensor system. This system was especially useful in the genotoxicity detection of Di(2-ethylhexyl) phthalate (DEHP), a rodent carcinogen, but negatively reported in most non-mammalian *in vitro* mutation assays, by providing a strong indication of genotoxicity for DEHP. A limitation for this biosensor system was that it might give false positive results in response to sodium butyrate and any other agents, which can trans-activate the *p21* gene in a *p53*-independent manner.

## Abbreviations

*p53*: a tumor suppressor
*p21*: cyclin-dependent kinase 1A inhibitor
CMV: cytomegalovirus
GFP: green fluorescent protein
eGFP: enhanced green fluorescent protein
HPRT: hypoxanthine-guanine phosphoribosyltransferase
TK: thymidine kinase
GADD: growth arrest and DNA damage
FG: flounder gill
G418: geneticin
DMSO: dimethyl sulfoxide
MTT: thiazolyl blue tetrazolium bromide
DEHP: di(2-ethylhexyl) phthalate
S9: induced rat liver microsomal activation system
MEM: minimal essential medium
BCS: bovine calf serum
FBS: fetal bovine serum
PBS: phosphate-buffered saline
MEF: mouse embryo fibroblast
LARII: Luciferase Assay Reagent II
RLU: relative light units
ECVAM: European Center for the Validation of Alternative Methods
LEC: lowest effective concentration

## 1. Introduction

The ocean is a final sink of environmental pollutants including genotoxicants, which are often transported into the sea by rainfall and rivers. Especially the accidental release of radioactive isotopes from nuclear power plants into the sea or river nearby has evoked more concern regarding the potential genotoxic effects on aquatic organisms. Fish species are an important population in the aquatic environment. It is desirable to develop fast and reliable fish bioassay systems to investigate the genotoxic effects of environmental pollutants on fish [1,2,3,4]. But the maintenance of fish in laboratory is costly and time-consuming, requiring specially designed aquarium facilities, water replacing, air aerating and feeding, *etc*. Especially for marine fish, the source of seawater is often a big problem [5,6]. *In vitro* cultured fish cell lines are a good alternative to living fish [7,8,9]. However, biosensor systems for genotoxicity detection based on genetically modified fish cells are lacking. 

Today, bacterial, yeast and mammalian cell-based bioassay systems have been developed and currently used in the battery of genotoxicity tests required by most regulatory authorities [10,11]. In bacteria, the Ames test is the most widely used assay, which is based on the reverse mutation of genetically modified bacterial strains [12]. It is fast and specific for the detection of gene mutations by chemicals or irradiation, but has a relatively low sensitivity and frequently fails to identify the genotoxic properties of some compounds and the mutagenic effects that depend on the cellular structures that are specific to eukaryotic cells [13]. The Umu and Vitotox tests are another kind of mutagenicity test developed for bacteria, which are based on the bacterial SOS response that is activated upon exposure to genotoxicants [14,15,16].

Several yeast-based genotoxicity assays have been developed to obtain genotoxicity data on eukaryotes. Like bacteria, some of them are based on reverse mutations in the genetically modified yeast strains [17,18,19]. But the others monitor the activation of DNA damage-induced over-expression of reporter genes including GFP (green fluorescent protein), eGFP (enhanced green fluorescent protein) and luciferase, which are driven by the promoter of DNA damage repair genes like *RAD54* [20,21,22]. And these yeast-based reporter gene assays show a high specificity and sensitivity in the genotoxicity detection of various compounds and are adaptable to high throughput screening [23,24,25,26]. 

Mammalian cell-based genotoxicity tests are more useful for risk assessment in humans. They are often designed to detect DNA damage, gene mutation or cellular DNA damage response. The Comet assay, widely used for the detection of DNA damage such as DNA strand breaks in mammalian cells, is rapid and sensitive but has a high rate of false positives [27,28,29]. Many mammalian cell-based gene mutation assays are available, but only four cell lines of Chinese hamster V97 and CHO cells, human lymphoblastoid TK6 cells, and mouse lymphoma L5178Y cells, and only three genetic loci of HPRT (hypoxanthine-guanine phosphoribosyltransferase), TK (thymidine kinase) and the cell membrane Na^+^/K^+^ ATPase gene, are well validated and widely used [30]. And low sensitivity is still a problem in these mammalian cell-based gene mutation assays. 

In mammalian cells, the transcription factor *p53* works as a guard keeper of the genome by inducing DNA damage repair, cell cycle arrest and apoptosis in response to cellular stresses leading to DNA damage, thus it is also called tumor suppressor. The DNA repair gene *P53R2*, which encodes a subunit of ribonucleotide reductase, is a *p53*-target gene activated in response to cellular DNA damage. Ohno *et al*. developed a *p53*R2-mediated luciferase reporter gene bioassay system for genotoxicity detection using human cells with wild-type *p53* [31]. Validation of this assay system indicated that it could be a rapid and reliable tool in the screening of genotoxic chemicals [32]. The growth arrest and DNA amage (GADD) gene of *GADD45a* is another *p53*-target gene in response to ionizing radiation [33]. Hastwell *et al*. established a GADD45a-mediated GFP reporter gene bioassay system for genotoxicity detection in human TK6 cells [34]. It was found that this assay system had both high specificity and high sensitivity in genotoxicity detection of different genotoxicants [35,36]. The cyclin-dependent kinase 1A inhibitor of *p21*^CIP1/WAF1^ is the major downstream target gene of activated *p53* and is responsible for causing cell cycle arrest following DNA damage [37,38]. Recently, Zager *et al*. reported the development of a *p21*-mediated eGFP reporter gene bioassay system for genotoxicity detection in human hepatoma HepG2 cells [39]. Challenging this, stably transformed HepG2 cells (p21HepG2GFP) by genotoxic agents with known mechanisms of action showed that this human cell biosensor system can be used for simple and fast detection of genotoxic agents.

Here, we describe the development and initial validation of a *p21*-mediated luciferase reporter gene bioassay system for genotoxicity detection using a continuous flounder gill cell line (FG). The FG cell line has been established and widely used to study the toxic effects and mechanisms of environmental pollutants on fish species [5,6,40,41,42,43,44,45]. Unlike mammalian cells, FG cells can be easily maintained in a wide range of temperatures from 15 °C to 30 °C. They grow well at room temperature and a sub-confluent monolayer can survive over one month at 15 °C without medium replacement [46]. This will provide an extraordinary merit in the shelf life and transportation once this fish cell biosensor system is marketed. The obtained results showed that this fish cell biosensor system could be used for rapid and high throughput screening of genotoxic agents. 

## 2. Experimental Section

### 2.1. Chemicals

Bleomycin sulfate, Mitomycin C, Geneticin (G418), Lipofectamine LTX and PLUS reagents were purchased from Invitrogen, USA. Dimethyl sulfoxide (DMSO), Thiazolyl blue tetrazolium bromide (MTT), Di(2-ethylhexyl) phthalate (DEHP) and Cyclophosphamide were obtained from Sigma, USA. D-Mannitol, Sodium butyrate and all the other reagents used were of analytical grade. 

The rat liver S9 mixture was prepared by combining 75 μL NADPH-RGS A solution, 15 μL NADPH-RGS B solution and 10 μL induced rat liver S9 male before use (Beijing Targin Tech Corporation, China).

The stock solution of DEHP (2 × 10^5^ μg/mL) was prepared by dissolving 0.1 mL 1 g/mL DEHP into 0.4 mL DMSO and then diluted into the working solutions using minimal essential medium (MEM) before use. All the other stock solutions of agents tested were prepared by direct dissolution in MEM.

### 2.2. Cell Line and Plasmids

The cell line FG was derived from the gill tissues of marine flatfish flounder (*Paralichthys olivaceus*) in 1993 and had since been maintained according to the method described by Tong *et al*. [46]. Briefly, the cells were cultured in MEM (Gibco, USA), supplemented with 10% bovine calf serum (BCS; Hyclone, USA), 100 IU/mL penicillin and 100 μg/mL streptomycin, in plastic cell culture flasks (Corning, USA) at 20 °C.

The expression plasmids of pGL_3_-p53-luc, pGL_3_-p21-luc and pRL-CMV were kindly provided by Xiongbin Lu (Department of Cancer Biology, University of Texas M.D. Anderson Cancer Center). Both pGL_3_-p53-luc and pGL_3_-p21-luc contain a firefly luciferase reporter gene, which was driven by the promoter of the human *p53* or *p21* gene, respectively. But the plasmid pRL-CMV contains a *Renilla* luciferase reporter gene driven by a constitutive promoter CMV (cytomegalovirus) and was used as internal reference control. A vector plasmid of pcDNA3.1/V5A-His (Invitrogen, USA) with a neomycin resistance gene was used in the co-transfection experiments of the reporter plasmids to provide the G418 resistance for the transformed FG cells.

### 2.3. Analysis of the Sensitivity of FG Cells to G418

FG cells were harvested and diluted to a concentration of 1 × 10^5^ cells/mL in MEM with 10% BCS. The cell suspension was agitated and 500 μL aliquots were added into each well of 24-well tissue culture plates. Plates were incubated overnight at 20 °C. The next day, the cells were re-fed with medium containing 0 (control), 100, 200, 300, 400, 500, 600, 700, 800, 900 and 1,000 μg/mL G418, respectively. The selective media were replenished every three to four days. The survival percentage of exposed FG cells was observed daily. The minimal concentration of G418 that can kill all the FG cells in two weeks was recorded and used for the selection of the stably transformed FG cells. Half of the obtained selection concentration of G418 was used in the maintenance of the stably transformed FG cells. 

### 2.4. Thiazolyl Blue Tetrazolium Bromide (MTT) Assay

The cytotoxic effects of bleomycin, mitomycin C and ethanol to FG cells were examined by MTT assay as described previously [5], which was based on the reduction of soluble yellow MTT tetrazolium salt to a blue insoluble MTT formazan product by mitochondrial succinate dehydrogenase. Briefly, 96-well tissue culture plates were seeded by 200 μL FG cell suspension (1.0 × 10^5^ cells/mL) per well and incubated at 20 °C for 24 h, then the medium was replaced with test medium containing varied concentrations of bleomycin, mitomycin C or ethanol (<40%). Alcohol is a kind of fixative. Higher concentrations of ethanol (>40%) will fix the FG cells and interrupt the use of the MTT method. After a 24 h exposure period, the test medium was replaced with 20 μL of 5 mg/mL MTT in phosphate-buffered saline (PBS; 3.0 g Na_2_HPO_4_·12H_2_O, 0.2 g KH_2_PO_4_, 8.0 g NaCl and 0.2 g KCl per liter water, pH 7.2) and incubated for 4 h at 20 °C. Then the MTT solution was carefully removed and the cells were rinsed twice with PBS rapidly. After that, 150 μL/well DMSO was added to dissolve the purple formazan crystals produced. Absorbance of each well was then measured at 490 nm and the 24h-IC_50_ values (the concentration of tested chemicals which causes a 50% inhibition compared to the control after a 24 h exposure period) were calculated by logistical regression analysis (Dose-response-Inhibition, log (inhibitor) *vs*. response-variable slope) using the GraphPad Prism 5 software.

All the experiments were performed in triplicate, and the mean absorbance at each concentration was calculated and expressed as the percentage of absorbance of the treated cells compared to the control. 

### 2.5. Validation of the Integrity of the Endogenous p53 Signaling Pathway in FG Cells

Whether or not the endogenous *p53* signaling pathway in FG cells works well was confirmed by examining the trans-activation of the expression of the human *p53* or *p21* promoter-driven luciferase reporter genes. The FG cells were seeded into six-well culture plates (1.5 mL cell suspension per well) and incubated for 1–2 days at 20 °C until the cells were 50%–80% confluent. The cells were first co-transfected with 1.5 μg/well plasmid DNA of pGL_3_-p53-luc (or pGL_3_-p21-luc), pRL-CMV and pcDNA3.1 at the ratio of 4:1:1, using Lipofectamine LTX and PLUS reagents (Invitrogen). In detail, plasmid DNA of 1 μg pGL_3_-p53-luc (or pGL_3_-p21-luc), 0.25 μg pRL-CMV and 0.25 μg pcDNA3.1 were mixed and diluted in 500 μL serum-free MEM; then 1.5 μL PLUS reagent was added into the diluted DNA, mixed gently and incubated for 5 min at room temperature; after that, 3 μL Lipofectamine LTX was added and the sample was mixed thoroughly; after 30 min incubation at room temperature, all the DNA-lipid complex was added drop-wise into the well and mixed gently by rocking the plate back and forth; the medium containing transfection reagents was replaced with regular medium after 4 h incubation at 20 °C.

**Figure 1:**
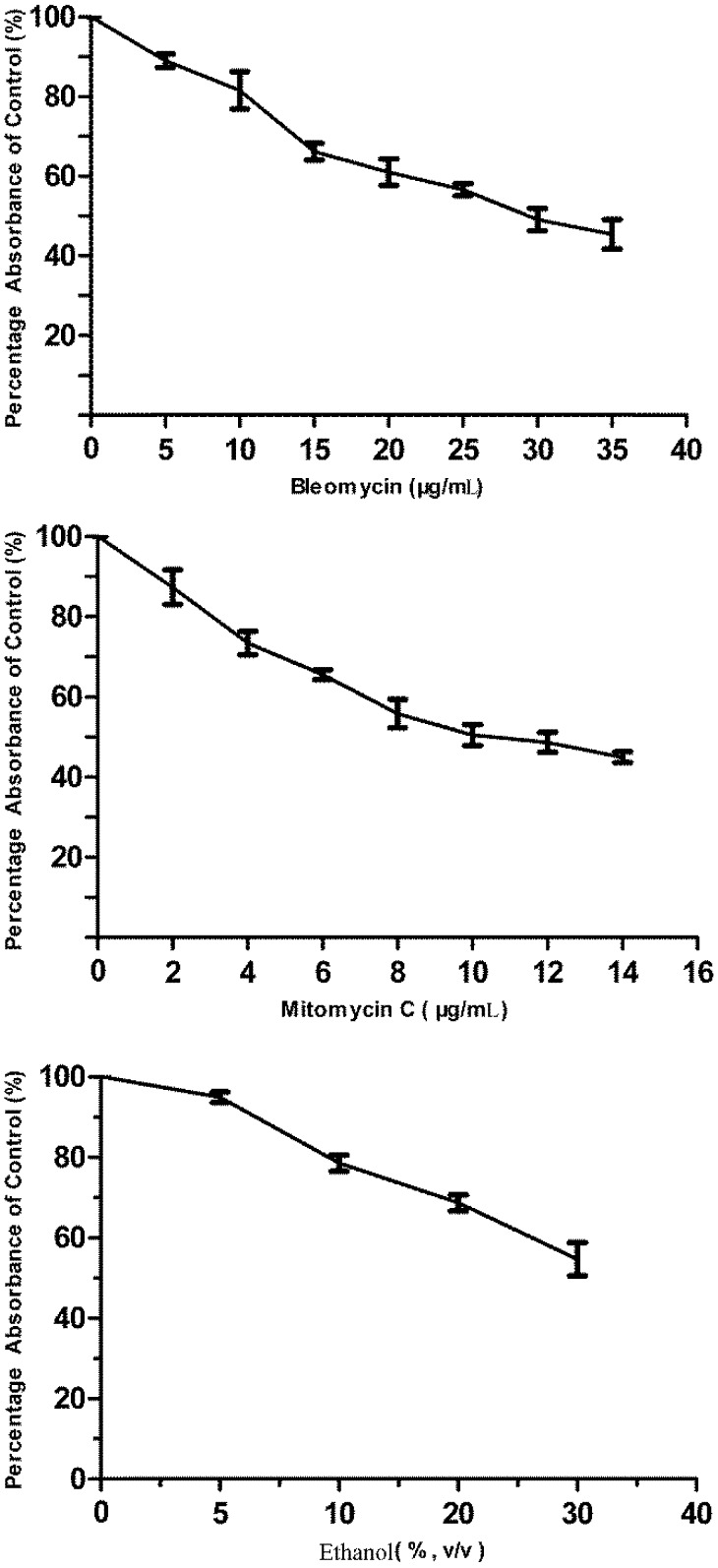
The cytotoxicity of bleomycin, mitomycin C and ethanol to flounder gill (FG) cells as determined by thiazolyl blue tetrazolium bromide *(*MTT) assay. Data are expressed as mean ± SD.

After two days’ recovery, the transiently transformed FG cells were then exposed to G418 selection for two weeks. At the end, the selective medium was replaced with regular medium and all the survival FG cells were then challenged with DNA damaging reagent of bleomycin (30 μg/mL) for 4 h. The above-mentioned dose and exposure time of bleomycin were selected for validation purposes, as they were high enough to initiate the *p53*-dependent DNA damaging response in mammalian cells [47,48,49], but imposed acceptable cytotoxicity on FG cells (Figure 1). After that, the challenged FG cells were collected and lysed. The activities of both firefly luciferase and *Renilla* luciferase of the obtained lysates were sequentially measured using the Dual-Luciferase^®^ Reporter Assay System (Promega, USA, Cat.#E1910) with a luminometer (GloMax^®^ 20/20, Promega, USA). The protocol for the dual reporter assay is presented in detail in Section 2.8. Intact FG cells (not transformed) were seeded and performed simultaneously as control. 

### 2.6. Construction of Stably Transformed FG Cells (p21FGLuc)

FG cells were co-transfected with three plasmids of pGL_3_-p21-luc, pRL-CMV and pcDNA3.1 at the ratio of 4:1:1 using Lipofectamine LTX and PLUS Reagents as described above. After two days of recovery, the transiently transformed FG cells were collected, diluted at 1:10 in fresh medium containing 10% fetal bovine serum (FBS; Hyclone, USA) and seeded into 24-well culture plates. Then the cells were exposed to G418 selection for more than four weeks. It was found that FG cells did not proliferate when seeded in a clone density or in an extremely low density. Thus the obtained G418-resistant FG cells were then pooled together and maintained under half level of G418 selection until used. Based on the results obtained in the sensitivity test of FG cells to G418, the final concentration of G418 in the selection medium for the stable p21FGLuc cells was 600 μg/mL and 300 μg/mL for maintenance of the same transgenic cells. 

In order to make up the failure of a traditional G418-mediated colonal selection due to cellular growth characteristics, the obtained stable p21FGLuc cells were subsequently passaged at a very low density under G418 selection for over six months and then the stability and level of the expression of the two reporter signals were examined. 

### 2.7. Challenging the p21FGLuc Cells with Model Genotoxins of Bleomycin and Mitomycin C and Non-Genotoxin of Ethanol

Bleomycin and mitomycin C are two model directly acting genotoxic agents with known mechanisms of action. Ethanol is reported as a non-genotoxic agent. These three agents imposed different cytotoxic effects on FG cells when examined by MTT assay. The obtained cytotoxicity data (Figure 1) showed that 30 μg/mL bleomycin, 10 μg/mL mitomycin C and 30% (v/v) ethanol produced similar cytotoxicity in FG cells with a survival percentage of 49%, 50% and 54% after 24 h exposure, respectively. Like bleomycin, the dose of 10 μg/mL mitomycin C was also selected for validation purpose, which was high enough to destroy the genomic DNA of mouse embryo fibroblast (MEF) cells after an exposure period of 2–3 h [50]. They are thus used in the challenge of p21FGLuc cells to examine the time course of cellular responses to genotoxic and non-genotoxic agents. 

To examine the time course of responses of the stable p21FGLuc cells to the agents tested, the p21FGLuc cells were collected and diluted to 1 × 10^5^ cells/mL, then 1 mL/well cell suspension was seeded into 24-well culture plates and incubated at 20 °C overnight. The next day, the old medium was removed and fresh medium containing 30 μg /mL bleomycin, or 10 μg /mL mitomycin C, or 30% (v/v) ethanol was added, respectively. After 1 h, 2 h, 3 h, 4 h and 5 h exposure of bleomycin, or 0.5 h, 1 h, 1.5 h and 2 h exposure of mitomycin C, or 1 h, 2 h, 3 h and 4 h exposure of ethanol, respectively, the treated p21FGLuc cells were lysed and the cell lysates were collected for dual luciferase reporter gene assay. 

The dose-dependent responses of the stable p21FGLuc cells to increasing concentrations of bleomycin, mitomycin C and alcohol were also examined in a similar way. The doses tested and exposure periods were 0 (control), 1, 5, 10, 15, 20, 25, 30 and 40 μg /mL for 4 h for bleomycin, 0 (control), 1, 2, 6, 10 and 14 μg /mL for 2 h for mitomycin C, and 0 (control), 1%, 5%, 10%, 15%, 20% and 30% (v/v) for 1 h for ethanol. The selection of optimal exposure times for the above-mentioned three agents was based on the results of previous time-course tests.

### 2.8. Dual Luciferase Reporter Gene Assay

The dual-luciferase reporter assay system provides an efficient way to perform the dual-reporter gene assay. In this assay, the activities of firefly (*Photinus pyralis*) and *Renilla* (*Renilla reniformis*) luciferases are measured sequentially from a single sample. The firefly luciferase reporter is measured first by adding Luciferase Assay ReagentII (LARII) to generate a stabilized luminescent signal. After quantifying the firefly luminescence, this reaction is quenched, and the *Renilla* luciferase reaction is simultaneously initiated by adding Stop & Glo Reagent to the same tube. The luciferase reporter gene assay of transformed FG cells was carried out according to the protocol from the manufacturer. Briefly, at ambient temperature, the medium of the tested cells in 24-well culture plates was removed and rinsed once in PBS. Then 100 μL/well of passive lysis buffer was added and the culture plate was shaken gently at room temperature for 15 min to ensure complete lysis. After that, all the cell lysate in each well was transferred into a 1.5 mL centrifuge tube. Then 20 µL of cell lysate was taken out and added into another 1.5 mL centrifuge tube containing 100 µL of LAR II, and mixed quickly by pipetting two or three times. Then the tube was immediately placed in the luminometer and the luminescence (relative light units, RLU) due to firefly luciferase activity was measured. After that, the sample tube was removed from the luminometer and 100 µL of Stop & Glo Reagent was added and mixed briefly, then the sample tube was put in the luminometer again, and the luminescence due to *Renilla *luciferase activity was recorded. 

The luminometer was programmed to perform a 2 s pre-measurement delay, followed by a 10 s measurement period for each reporter assay. The responsive capacities of the stable p21FGLuc cells to genotoxins or non-genotoxins were indicated as and normalized by the ratio of the RLU value of firefly luciferase to the RLU value of *Renilla* luciferase.

### 2.9. Challenging the p21FGLuc Cells with Varied Doses of Cyclophosphamide, Di(2-ethylhexyl) Phthalate (DEHP), D-Mannitol and Sodium Butyrate

As recommended in the ECVAM (European Center for the Validation of Alternative Methods) list [51], cyclophosphamide is a kind of carcinogen that exerts its genotoxicity on cells only after metabolic activation, and the plasticizer of Di(2-ethylhexyl) phthalate (DEHP) is classified as a rodent carcinogen and is negatively reported in most non-mammalian *in vitro* mutation assay, and D-mannitol is a model non-genotoxin. To further validate the specificity and sensitivity of p21FGLuc cells in response to the indirectly-acting *in vivo* genotoxin of cyclophosphamide, non-genotoxic carcinogen of DEHP, and non-genotoxin of D-mannitol, the p21FGLuc cells were exposed to 0 (control), 3.13, 6.25, 25 and 50 μg/mL cyclophosphamide with or without the metabolic activation of rat liver S9 mixture, or to 0 (control), 0.005, 0.05, 0.5, 5, 50 and 200 μg/mL DEHP, or to 7.1, 56.9, 455.5, 911, 1,822 μg/mL D-mannitol, respectively. The above-mentioned dose selection was based on the lowest effective concentration (LEC) results by GADD45a-GFP GreenScreen HC assay [52] and for DEHP, the environmental concentration was included.

After 24 h exposure, the treated p21FGLuc cells were lysed and the cell lysates were collected for dual luciferase reporter gene assay as described previously. In the metabolic activation of cyclophosphamide, the medium containing varied doses of cyclophosphamide was supplemented with 10% S9 mixture and incubated at 37 °C for 30 min before the cyclophosphamide-containing medium was added into the cells. 

The up-regulation of *p21* expression can be induced by both *p53*-dependent and *p53*-independent mechanisms [53]. Butyrate is a short chain fatty acid and reported to be able to activate *p21* transcription independent of the DNA damage-induced *p53*-signaling pathway [54]. Therefore, we also examined the cellular response of p21FGLuc cells to the exposure of 0 (control), 0.1, 1, 2, 5 mM sodium butyrate for 24 h, as described previously.

### 2.10. Statistical Analysis

All the experiments were conducted at least three times. Statistical analyses were performed using the program GraphPad Prism 5 (GraphPad software, Inc., San Diego, USA). The statistical significance of the difference between mean values was determined by ANOVA, and a difference with p < 0.05 was considered significant. All data were expressed as mean ± standard deviation (SD).

## 3. Results

### 3.1. Cytotoxicity of Bleomycin, Mitomycin C and Ethanol to FG Cells

As shown in Figure 1, all three agents tested showed dose-dependent cytotoxicity in FG cells. Mitomycin C was much more toxic to FG cells than bleomycin. The IC_50_ values for bleomycin, mitomycin C were 37.80 ± 1.46 μg/mL and 14.57 ± 1.28 μg/mL, respectively. However, we did not obtain the corresponding IC_50_ value for ethanol. It was shown that 30 μg/mL bleomycin, 10 μg/mL mitomycin C and 30% (v/v) alcohol produced similar cytotoxicity in FG cells with a survival percentage of 49%, 50% and 54% after 24 h exposure, respectively.

### 3.2. The Sensitivity of FG Cells to G418

**Figure 2:**
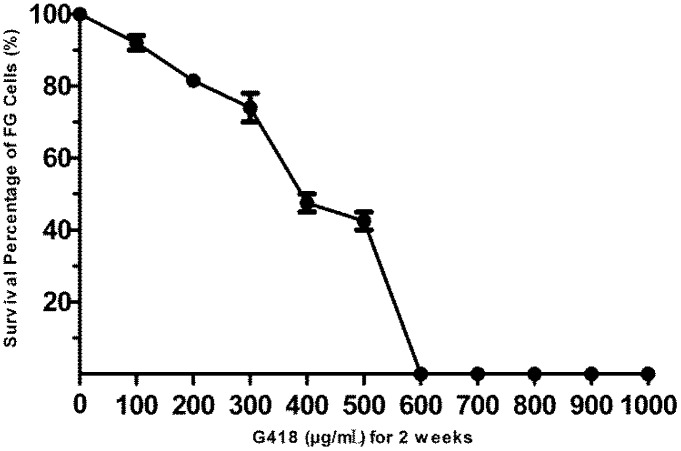
The sensitivity of FG cells to G418. FG cells were exposed to increasing concentrations of G418 for two weeks and the survival percentage of treated cells to untreated cells is calculated. Data are expressed as mean ± SD.

As shown in Figure 2, G418 imposed obvious cytotoxic effects on FG cells in a dose-dependent manner. The lowest concentration of G418 that can kill all the exposed FG cells after a two-week exposure period was 600 μg/mL. Thus the G418 dose of 600 μg/mL was chosen in the following selection for the stably transformed FG cells, and 300 μg/mL G418 was used in the maintenance of stably transformed FG cells. 

### 3.3. FG Cells Have Wild-Type p53 Signaling Pathway

**Figure 3:**
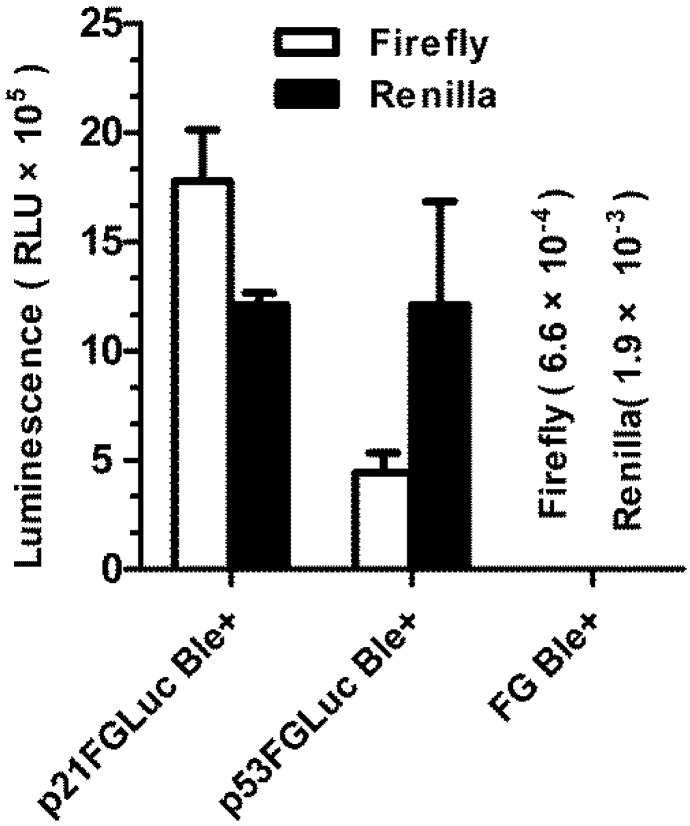
Validation of the endogenous *p53*-signaling pathway in FG cells. Examination of the responses of the transiently transformed FG cells to genotoxicant of bleomycin (30 μg/mL for 4 h) using firefly luciferase reporter plasmids of pGL_3_-p21-luc and pGL_3_-p53-luc and the *Renilla* luciferase internal reference plasmid of pRL-CMV. The intact FG cells (not transformed) were used as control. Data are expressed as mean ± SD.

As shown in Figure 3, the DNA damaging agent of bleomycin can significantly induce the over-expression of both the *p53* and *p21* promoter-driven firefly luciferase reporter genes in the transiently transformed FG cells. The efficient trans-activation of pGL_3_-p53-luc plasmid by DNA damage-induced *p53* signaling pathway of FG cells indicates that the upstream regulators of the p53 protein in response to DNA damage are valid in FG cells. Similarly, the efficient trans-activation of pGL_3_-p21-luc plasmid showed that FG cells still held wild-type p53 proteins in spite of the fact that they had become a continuous cell line and undergone neoplastic transformation, and also p53 proteins of FG cells were able to recognize and trans-activate the promoter of human *p21* gene. In contrast, extremely low background luciferase activity was detected in the untransformed FG cells. The luminescence of firefly luciferase and *Renilla* luciferase in the untransformed FG cells was only 66 RLU and 190 RLU, respectively, about eight to nine orders of magnitude lower than those in the transformed FG cells. It was also observed that the expression of *Renilla* luciferase from pRL-CMV plasmid was not DNA damage-inducible but cell number-dependent (data not shown). 

### 3.4. Construction of Stably Transformed FG Cells (p21FGLuc)

**Figure 4:**
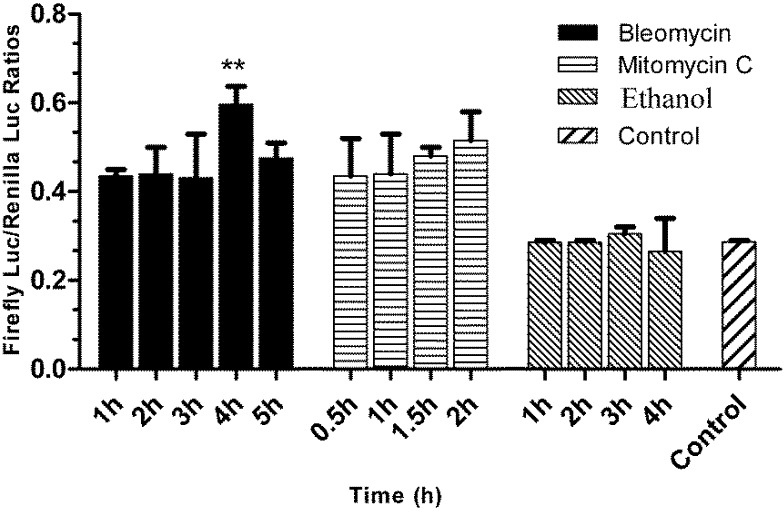
The time-course responses of the stable p21FGLuc cells to model genotoxic and non-genotoxic agents. The stable p21FGLuc cells were exposed to 30 μg /mL bleomycin, 10 μg /mL mitomycin C and 30% (v/v) ethanol, respectively. The same stable p21FGLuc cells, not exposed to any toxicants but with the same volume of PBS, were used as control. ** Shows the highly significant difference (p < 0.01). Data are expressed as mean ± SD.

**Figure 5:**
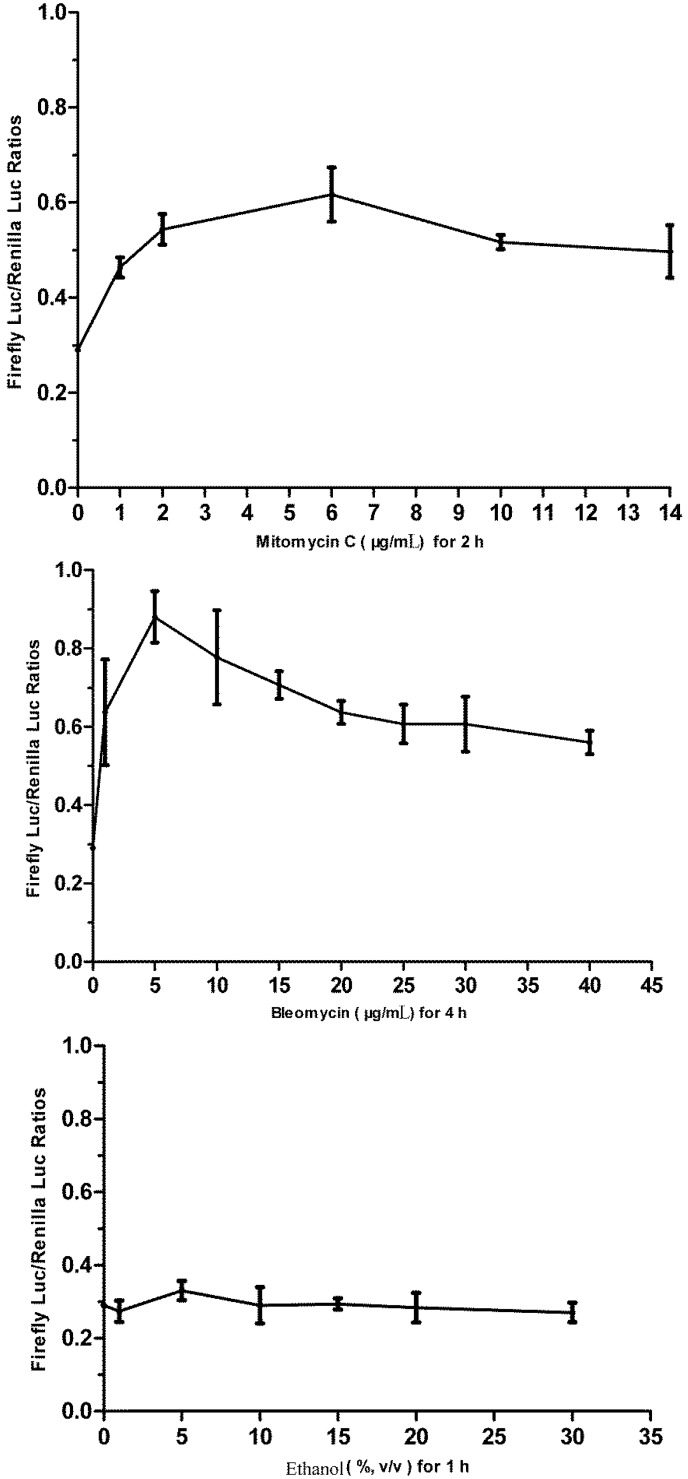
The dose-dependent responses of the stable p21FGLuc cells to model genotoxic and non-genotoxic agents. The stable p21FGLuc cells were exposed to increasing concentrations of bleomycin, mytomycin C and ethanol. Data are expressed as mean ± SD.

**Figure 6:**
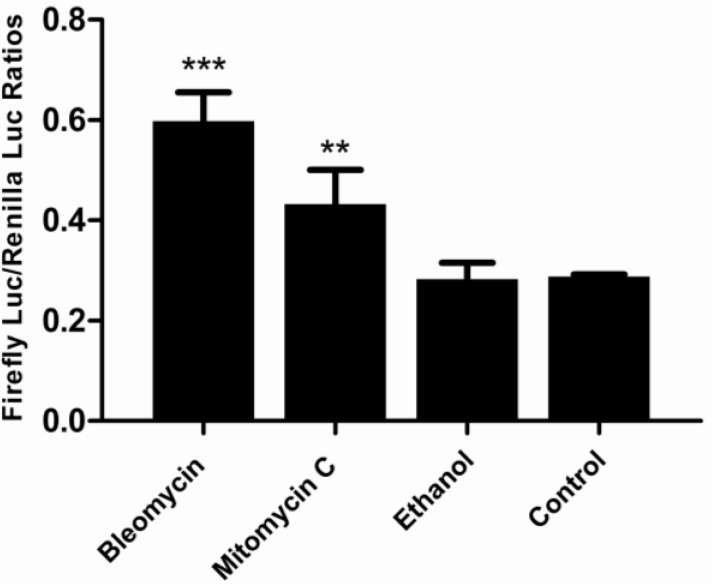
Comparison of the DNA damage-induced responses of the stable p21FGLuc cells to bleomycin, mitomycin C and ethanol. The p21FGLuc cells were exposed to 30 μg/mL bleomycin for 4 h, 10 μg /mL mitomycin C for 2 h and 30% (v/v) ethanol for 1 h, respectively. ** Shows the highly significant difference (p < 0.01). *** Shows the very highly significant difference (p < 0.001). Data are expressed as mean ± SD.

FG cells have been successfully co-transfected with three expression plasmids of pGL_3_-p21-luc, pRL-CMV and pcDNA3.1. As shown in Figure 4, Figure 5 and Figure 6, the obtained stable p21FGLuc cells have a constant basal level of firefly luciferase and *Renilla* luciferase expression even after one year of selection and maintenance with G418. Challenge of genotoxicants of bleomycin and mitomycin C can significantly up-regulate the expression of firefly luciferase in the stable p21FGLuc cells. 

### 3.5. Responses of the p21FGLuc Cells to the Exposure of Model Genotoxic and Non-Genotoxic Agents

To examine the specificity and sensitivity of the obtained fish cell biosensor system for detection of genotoxic agents, we tested the time-dependent and dose-dependent responses of the obtained p21FGLuc cells to model genotoxic and non-genotoxic agents. 

As shown in Figure 4, the challenge of the p21FGLuc cells with the two genotoxic agents of bleomycin (30 μg/mL) and mitomycin C (10 μg/mL) significantly up-regulated the expression of the firefly luciferase in the treated cells in contrast to the untreated cells, but the challenge with non-genotoxic agent of ethanol (30%, v/v) did not induce the up-regulated expression of the firefly luciferase in the treated cells compared with the untreated cells. And also, the response speed of this biosensor system to genotoxic agents was fast and the induced expression level of reporter genes was relatively stable among the different exposure periods tested. For bleomycin, the DNA damage-induced response was detected after 1 h of exposure, and a similar expression level of reporter genes was obtained after 5 h of exposure although it reached a peak value at 4 h. For mitomycin C, the induced response was detected after a 30 min exposure and slightly up-regulated after a 2 h exposure. 

However, this fish cell biosensor system showed an obvious dose-dependent response to genotoxic agents but not to the non-genotoxic agent (Figure 5). In detail, when exposed to bleomycin at a concentration lower than 5 μg/mL, the p21FGLuc cells produced more firefly luciferase with an increasing dose of bleomycin; however, when the concentration of bleomycin was higher than 5 μg/mL, the production of firefly luciferase by the p21FGLuc cells decreased with the increase of the dose of bleomycin. Similar dose-dependent responses were observed in the challenge of the p21FGLuc cells with increasing concentrations of mitomycin C except for the curve inflection point corresponding to 6 μg/mL mitomycin C. In addition to a clear dose- and time-effect responsive relationship between the exposure of genotoxins and the trans-activation of *p53*-signaling pathway, the above-mentioned results also inferred that cytotoxicity of genotoxic agents in the p21FGLuc cells may counterbalance their corresponding genotoxic effects to some degree. 

Under similar cytotoxicity of the three toxicants tested to the p21FGLuc cells, bleomycin exhibited higher genotoxicity than mitomycin C, but alcohol showed no obvious genotoxicity (Figure 6). 

### 3.6. Responses of the p21FGLuc Cells to Varied Doses of Cyclophosphamide, Di(2-ethylhexyl) Phthalate (DEHP), D-Mannitol and Sodium Butyrate

**Figure 7:**
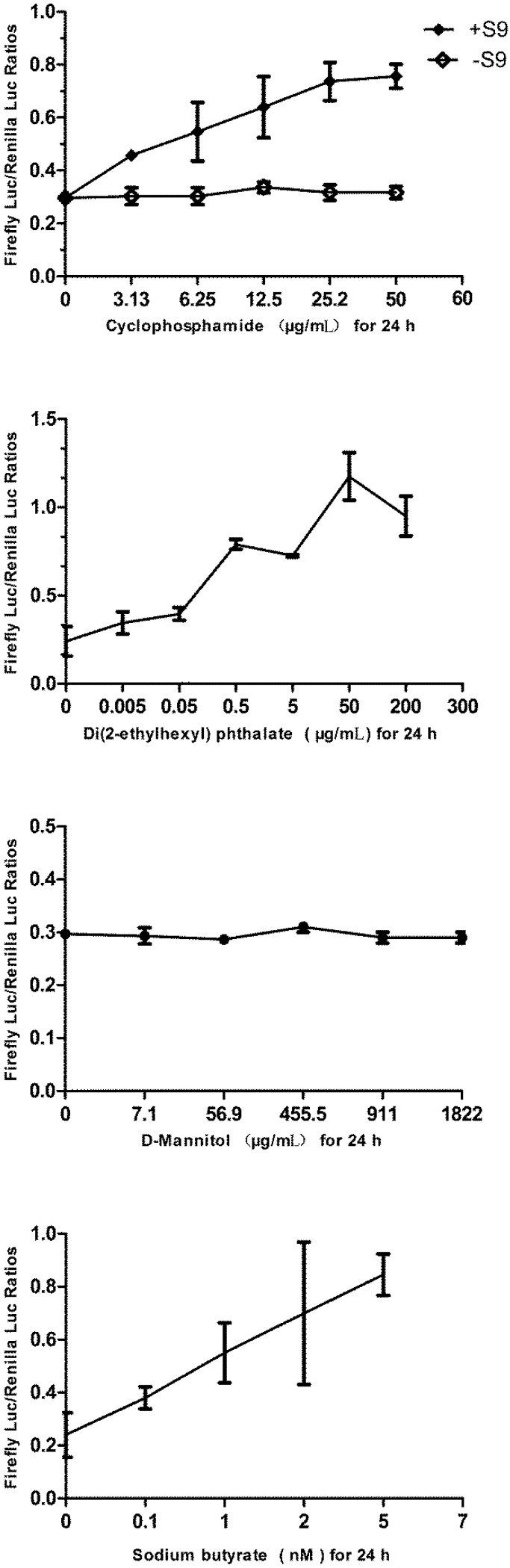
The responses of the p21FGLuc cells to varied concentrations of cyclophosphamide, di(2-ethylhexyl) phthalate (DEHP), D-mannitol and sodium butyrate for 24 h. S9+ and S9− refer to results with and without metabolic activation by the S9 mixture, respectively. Data are expressed as mean ± SD.

As shown in Figure 7, after metabolically activated by rat liver S9 mixture, cyclophosphamide could significantly induce the up-regulation of the expression of firefly luciferase in the exposed p21FGLuc cells in a dose-dependent manner, but not for the cyclophosphamide without S9 mixture pretreatment. Similar positive responses were observed in the p21FGLuc cells exposed to increasing concentrations of DEHP. Even at an environmental dose level of 0.005 μg/mL DEHP, obvious up-regulation of the expression of firefly luciferase was observed in the exposed p21FGLuc cells. At the concentration of 50 μg/mL DEHP, the induced expression level of firefly luciferase in the exposed cells reached a peak level. In contrast, D-mannitol did not induce any up-regulation of firefly luciferase expression in the exposed cells like ethanol. 

It is noteworthy to mention that sodium butyrate could also induce the up-regulation of firefly luciferase expression in the exposed p21FGLuc cells (Figure 7). But it is quite different from the curves obtained by genotoxic agents of bleomycin, mitomycin C, cyclophosphamide and DEHP, as the induced over-expression of firefly luciferase in the exposed cells had a positive linear relationship with the doses of sodium butyrate added.

## 4. Discussion

### 4.1. A Prerequisite for FG Cells to Be Engineered into a Genotoxicity Biosensor System Is to Hold a Wild-Type p53 Signaling Pathway

In mammalian cells, the integrity and validity of the *p53*-signaling pathway is indispensible for the maintenance of genomic stability [55,56]. In unstressed cells, *p53* is unstable and present at extremely low levels because of rapid degradation by Mdm2 (mouse double minute 2). In stressed cells, however, the DNA damage signal from double or single-strand breaks can be recognized by ATM (ataxia telangiectasia mutated) or ATR (ataxia telangiectasia and Rad3-related protein) kinases, respectively, and they are then activated and further activate the checkpoint kinases of Chk1 and Chk2 by phosphorylation. The above-mentioned activated kinases then phosphorylate the *p53* protein and its negative regulator Mdm2. This phosphorylation results in stabilization and increased levels of *p53*. The accumulated *p53* will trans-activate a series of downstream target genes involved in cell cycle arrest, DNA repair and apoptosis. Thus *p53* protects the cells from propagating mutations induced by DNA damage by arresting the cells in G1 phase, allowing time for DNA damage to be repaired prior to re-entry into S phase. Alternatively, if the damage is too extensive or the cell is comprised in a dangerous way (e.g., cancerous), apoptosis is induced. Either way, the organism is protected from cells acquiring oncogenic lesions [57,58]. 

However, it was found that *p53* is the most frequently mutated tumor suppressor gene in mammalian tumors [59]. Over half of the human tumors have a mutated *p53* gene, and many of the remaining tumors have defects in *p53* signaling pathways. This greatly hampers the use of genetically modified mammalian cells for genotoxicity detection based on the *p53*-signaling pathway because most mammalian cell lines are derived from tumor tissues. In contrast, most of the established fish cell lines are derived from normal fish tissues. The FG cell line used in the present study was established from normal gill tissues of flounder in 1993 [46] and had undergone neoplastic transformation [60]. But we showed in this study that FG cells still preserve the wild-type *p53*-signaling pathway in that both of the two luciferase reporter plasmids driven by the promoters of *p53* and *p21*, respectively, can be trans-activated effectively (Figure 3). This is a prerequisite for FG cells to be engineered into a genotoxicity biosensor system based on DNA damage-induced trans-activation of target genes. 

### 4.2. Flounder p53 Protein Can Effectively Recognize and Trans-Activate the Expression of Human p21 Gene

Orthologs of the *p53* gene have been reported in many non-mammals including clams [61,62], squid [63], flies [64], frogs [65] and zebrafish [66]. However, it seemed that only zebrafish *p53* held the capacity to trans-activate its downstream target of *p21* gene in response to DNA damage [67]. And also, like mammals, zebrafish Mdm2 protein negatively regulated the level of p53 protein [68], but not in the same way as in invertebrates. 

Sequence analysis has shown that the *p53* gene is structurally conserved from invertebrates to mammals, containing three domains corresponding to transcriptional activation, DNA binding and tetramerization activity. Of these domains, the DNA binding domain, which is responsible for trans-activation of downstream target genes, is the most conserved domain [67]. Consistent with this, it was found in this study that flounder p53 protein could effectively recognize the promoter of the human *p21* gene and trans-activate the expression of the fused firefly luciferase gene (Figure 3).

In the time-course test of bleomycin exposure in the p21FGLuc biosensor system, as shown in Figure 4, the DNA damage-induced up-regulation of reporter gene based on the human *p21* promoter response was detected after 1 h exposure, and reached a peak value at 4 h. This time-dependent response of p21FGLuc cells to bleomycin indicated that the DNA damage-induced up-regulation of endogenous flounder p53 protein and the subsequent trans-activation of human *p21* promoter were also time-dependent. Similar responses were observed in previous western blot work, where it was found that the significant increase of the level of p53 protein began after 2 h exposure of U2OS cells (*p53*+) to bleomycin (data not published). It can be hypothesized that at least another two hours are needed to fully trans-activate the expression of the *p21* gene. Therefore, it was not strange in the present study to observe the significantly higher response to bleomycin after a 4 h exposure.

### 4.3. Initial Validation of the Fish Cell Biosensor System for Genotoxicity Detection

Here, we reported the development of a fish cell biosensor system for genotoxicity detection by the stable integration of three expression plasmids of pGL_3_-p21-luc (*p21* promoter linked to firefly luciferase gene), pRL-CMV (CMV promoter linked to *Renilla* luciferase gene) and pcDNA3.1 into FG cells. In this biosensor system, two reporter genes were introduced, and they were simultaneously expressed and measured sequentially within a single test system. The expression of firefly luciferase is correlated with the DNA damage response to genotoxicants, while the expression of *Renilla* luciferase serves as an internal control, normalizing the experimental variability caused by differences in cell viability or extraneous influences in dual-reporter assays including pipetting volumes, cell lysis efficiency and assay efficiency, thus allowing more reliable data to be obtained from this fish cell biosensor system in comparison with the single luciferase reporter systems [31]. 

The results for the initial validation of this fish cell biosensor system for genotoxicity detection are summarized in Table 1. First, two model genotoxic agents with known mechanisms of action and one non-genotoxic agent were selected to challenge the obtained p21FGLuc cells to investigate the ability of this biosensor system. Mitomycin C is a potent DNA cross linker. A single crosslink per genome has shown to be effective in killing bacteria. And exposure to 10 μg/mL mitomycin C for 3 h can effectively block the mitosis of mouse embryonic fibroblast (MEF) cells [50,69]. Bleomycin is a reactive oxygen species generator. It induces DNA strand breaks by producing superoxide and hydroxide free radicals that cleave DNA [70]. Ethanol is a central nervous system depressant and has significant psychoactive effects in sub-lethal doses [71]. It produces negative results in all the *in vitro* bacterial, yeast and mammalian cell-based genotoxicity assays, as well as in the *in vivo* micronucleus and chromosome aberration tests except for rodent carcinogenicity [34], indicating that ethanol may induce tumors in rodents in a non-genotoxic way. The results obtained in the challenging experiments show that (1) the obtained p21FGLuc cells can significantly up-regulate the expression of the firefly luciferase reporter gene in response to the DNA damage induced by the two genotoxic agents bleomycin and mitomycin C, but not in response to the ethanol stress; (2) the response speed of this biosensor system to genotoxic agents is fast and the induced expression level of reporter genes is relatively stable among the different exposure periods tested; (3) the response of this fish cell biosensor system to genotoxic agents is dose-dependent, but not in the case of the non-genotoxic agent; (4) under similar cytotoxicity, bleomycin exhibits higher genotoxicity to the p21FGLuc cells than mitomycin C. In short, it is suggested that this fish cell biosensor system may become a rapid and reliable tool for genotoxicity detection.

**Table 1:** Summary of the genotoxicity detection results using the fish cell biosensor system (p21FGLuc).

Chemicals		Genotoxicity Results	Concentration Tested	Exposure Time
Direct activators				
	Bleomycin	positive	1–40 μg/mL	4 h
	Mitomycin C	positive	1–14 μg/mL	2 h
Indirect activators				
Cyclophosphamide		Negative (without S9)	3.13–50 μg/mL	24 h
		Positive (with S9)	3.13–50 μg/mL	24 h
Alternate toxicants				
	Di(2-ethylhexyl) phthalate (DEHP)	positive	0.005–200 μg/mL	24 h
Unreactive activators				
	Ethanol	negative	1%–30%	1 h
	D-mannitol	negative	7.1–1,822 μg/mL	24 h
P21 direct activators				
	Sodium butyrate	false positive	0.1–5 nM	24 h

To reduce falsely predictive positive or negative results, it is vital for this fish cell biosensor system to have high specificity and sensitivity in the genotoxicity detection of new chemicals and drug development as well as environmental monitoring. In the present study, results obtained in the evaluation tests using varied concentrations of cyclophosphamide, DEHP and D-mannitol further confirmed the specificity and sensitivity of this fish cell biosensor system in the genotoxicity detection (Table 1). 

Cyclophosphamide is carcinogenic and shows positive results in the Ames test, *in vivo* genotoxicity tests and *in vitro* mammalian cell tests but requires metabolic activation [51]. It is converted in the liver by oxidase enzymes of microsomes to active metabolites of phosphoramide mustard. Phosphoramide mustard forms DNA cross-links both between and within DNA strands at guanine N-7 positions. This is irreversible and leads to cell death [72]. Challenge of p21FGLuc cells with varied doses of cyclophosphamide after metabolic activation also showed positive results, but negative results for cyclophosphamide without metabolic activation.

Di(2-ethylhexyl) phthalate (DEHP), a commonly used plasticizer, is one of the most widespread environmental pollutants in the world [73]. DEHP is a rodent carcinogen and often reported to be negative in most of the bacterial, yeast and mammalian cell-based *in vitro* mutation assays. However, a number of *in vitro* rodent tissue assays have reported DEHP to be positive for effects on chromosomes, spindle, and mitosis, and also *in vitro* exposure of human cells or tissues to DEHP-induced DNA damage [74]. It is noteworthy that DEHP showed positive results in the present fish cell biosensor system for genotoxicity detection. This is a strong indication of the genotoxicity of DEHP, but the specific mechanism of action is still unclear. Moreover, at the environmental dose (0.005 μg/mL), DEHP obviously induced a DNA damage response in p21FGLuc cells; at 50 μg/mL, the DNA damage response went to a peak. In this case, the p21FGLuc biosensor system was more sensitive than other genotoxicity bioassay systems.

D-mannitol is a model non-genotoxin and shows negative results in all known *in vitro* and *in vivo* genotoxicity tests. In agreement with this, the present fish cell biosensor system for genotoxicity detection showed negative results, too.

The ability of the obtained p21FGLuc biosensor system for genotoxicity detection is based on the *p53*-dependent DNA damage response. However, it was reported that up-regulation of *p21* could also be *p53*-independent [53]. Butyrate has been shown to induce p21 expression via the Sp1-3 site in the *p21 *promoter independent of *p53* [54]. Similar results were obtained in this study. Sodium butyrate could induce the up-regulation of firefly luciferase expression in the exposed p21FGLuc cells (Figure 7). But it is quite different from the curves obtained by the genotoxic agents bleomycin, mitomycin C, cyclophosphamide and DEHP, and the induced over-expression of firefly luciferase in the exposed cells had a positive linear relationship to the doses of sodium butyrate added. This feature can serve to reduce the false positive results for p21FGLuc cells. In any case, this is a limitation for the p21FGLuc cell biosensor system. That is, p21FGLuc cells give a false positive result in response to sodium butyrate and any other agents, which can trans-activate the over-expression of *p21* gene in a *p53*-independent manner. 

In addition, we also obtained stably transformed p53FGLuc cells and tried to develop another fish cell biosensor system for genotoxicity detection based on the trans-activation of *p53* gene expression. However, the stable *p53*FGLuc cells did not survive and underwent apoptosis during the further maintenance (data not shown). A possible explanation for this is that the signals, which trans-activated the reporter plasmid of pRL_3_-p53-luc, also trans-activated the over-expression of the endogenous p53 protein, which led to the apoptosis of p53FGLuc cells. This makes it necessary to establish another biosensor system based on the trans-activation of the *p53* gene in *p53*^−/−^ cells in order to make up the limitation of the p21FGLuc cell biosensor system. 

In short, we still believe that this fish cell biosensor system may become a specific and sensitive tool for genotoxicity detection of new chemicals and drugs. In addition, the obtained p21FGLuc cells can be easily maintained at room temperature and a sub-confluent monolayer can survive over one month at 15 °C without medium replacement. This will facilitate the marketing of this fish cell biosensor system.
